# EHMTI-0119. Increased prevalence of migraine in patients with unruptured saccular intracranial aneurysms (SIA)

**DOI:** 10.1186/1129-2377-15-S1-G13

**Published:** 2014-09-18

**Authors:** HD Hambardzumyan, HM Manvelyan, GA Avagyan, HH Hakobyan, IM Gabrielyan

**Affiliations:** 1 Neurology, Yerevan State Medical University, Yerevan, Armenia

## Introduction

Rupture of SIA causes thunderclap headache but it remains unclear whether headache in general and migraine in particular is more prevalent in patients with unruptured SIA.

## Aims

In this case-control study we therefore estimate the prevalence of headaches in patients with SIA during 1 year before rupture.

## Methods

Prospectively 155 consecutive patients with SIA(96 women and 59 man, mean age 45.4 years) and 184 healthy blood donors (98 men, 86 women mean age 39.6 years) received o purpose developed semistructured interview. Diagnosis were made according to the International Headache Society criteria. Aneurysms were diagnosed by conventional cerebral angiography.

## Results

Headaches in patients with SIA before their diagnostics or rupture were revealed in 103 patients, therefore their 1-year prevalence was 61.6%. The mean duration of these headaches was 12.5 years, the mean age at the beginning of headaches was 30.2 years. These headaches included:migraine without aura(MO)-58(40.2%), migraine with aura(MA)-2(1%),tension type headache (TTH)-19(18.4%), cluster headache(CH)-2(1%), post-traumatic headaches(PH)-2(1%).1-year prevalence of headaches in controls was 32.5%(58 patients out of 184), they included:TTH-41(23.2%), MO-16(8.8%), PH-1(0.5%). Among this headaches in patients with SIA and controls only the prevalence of migraine was significantly (4.5times) higher in patients with SIA (OR 4.5, 95% CI 2.5-7.8, p<0.0001).

## Conclusions

This is the first study that convincingly shows a significant association between unruptured SIA and migraine.

No conflict of interest.

